# Current and future role of CT and advanced CT applications in inflammatory arthritis in the clinic and trials

**DOI:** 10.1007/s00256-025-04931-4

**Published:** 2025-04-16

**Authors:** Torsten Diekhoff, Sevtap Tugce Ulas

**Affiliations:** 1 Department of Radiology, Brandenburg Medical School, Rüdersdorf, Germany; 2 Department of Radiology, Charité - Universitätsmedizin Berlin, Humboldt-Universität Zu Berlin, FreieUniversität Berlin, Campus Mitte, Berlin, Germany; 3 Department of Radiology, Immanuel Klinik Rüdersdorf, Seebad 82/83, 15562 Rüdersdorf Bei Berlin, Germany

**Keywords:** Computed tomography, Arthritis, Gout, Axial spondyloarthritis

## Abstract

Computed tomography (CT) has traditionally been underutilized in the imaging of inflammatory arthritis due to its limitations in assessing soft tissue inflammation and concerns over radiation exposure. However, recent technological advancements have positioned CT as a more viable imaging modality for arthritis, offering high specificity and sensitivity in detecting structural bone changes. However, advances in ultra-low-dose CT protocols and AI-driven image reconstruction have significantly reduced radiation exposure while maintaining diagnostic quality. Dynamic CT and spectral CT techniques, including dual-energy CT (DECT), have broadened CT’s application in assessing dynamic joint instabilities and visualizing inflammatory changes through material-specific imaging. Techniques such as CT subtraction imaging and iodine mapping have enhanced the detection of active soft-tissue inflammation, virtual non-calcium reconstructions, and the detection of bone marrow edema. Possible CT applications span various forms of arthritis, including gout, calcium pyrophosphate deposition disease (CPPD), psoriatic arthritis, and axial spondyloarthritis. Beyond its diagnostic capabilities, CT’s ability to provide detailed structural assessment positions is a valuable tool for monitoring disease progression and therapeutic response, particularly in clinical trials. While MRI remains superior for soft tissue evaluation, CT’s specificity for bone-related changes and its potential for integration into routine arthritis management warrant further exploration and research. This review explores the current and emerging roles of CT in arthritis diagnostics, with a focus on novel applications and future potential.

## Introduction

Since its inception, computed tomography (CT) has been utilized to evaluate cortical and trabecular bone, as it directly visualizes and quantifies materials with relatively high electron density, such as hydroxyapatite within calcified bone. Due to its rapid scanning capabilities, broad availability, and superior spatial resolution, CT is typically the first cross-sectional imaging technique considered in emergency situations, such as when evaluating fractures [1]. However, CT has never achieved the status of a first-line imaging modality for arthritis due to several limitations, especially when compared to magnetic resonance imaging (MRI) or ultrasonography [2].

CT is characterized by low soft-tissue contrast, making it challenging to assess soft-tissue inflammation, such as synovitis in peripheral joints, as well as bone marrow edema. Additionally, rheumatologists are often concerned about the radiation exposure associated with conventional CT, particularly for the axial skeleton in younger patients, as it exceeds the exposure from standard radiographs. The inability to assess active inflammation, combined with a relatively high radiation burden, makes conventional CT suboptimal for routine arthritis imaging, confining its use to specific situations—primarily when MRI or ultrasound is unavailable or when crystal arthropathy is suspected [3].

Nevertheless, CT’s high diagnostic accuracy for detecting differential diagnoses is an often overlooked strength [4, 5]. With recent advancements in CT technology, such as ultra-low-dose protocols, higher spatial resolution, motion-sensitive dynamic imaging, and spectral information, CT may become more relevant in arthritis imaging in the future. This article aims to review recent and future developments in CT imaging for assessing arthritis in the peripheral and axial skeleton, as well as its potential applications in clinical settings and clinical trials.

## Technical aspects

### Ultra-low-dose CT

**Fig. 1:**
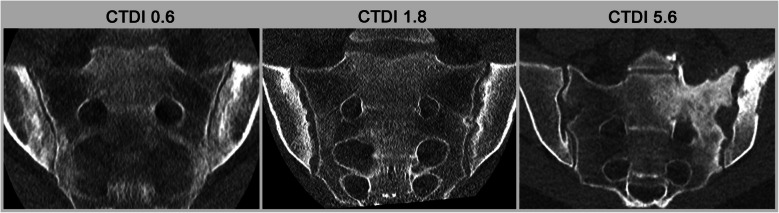
Comparison of ultra-low-dose (ULD)-CT, low-dose (LD-)CT, and standard CT for sacroiliitis. The three patients present with comparable age and weight. Images for ULD-CT and LD-CT were reconstructed with artificial intelligence, while the standard CT used iterative reconstructions. In all patients, structural lesions compatible with sacroiliitis are clearly visualized. While ULD-CT utilizes only 10% radiation of the standard CT, it also shows some degradation in image quality with loss of clear delineation of the cortical bone. CTDI is presented as mGy

CT imaging of bone is particularly well suited for dose reduction due to the high contrast between bone and surrounding soft tissues, which allows for tolerance of significant image noise. Consequently, diagnostic-quality imaging can be achieved even with standard reconstruction methods such as filtered back projection. Modern CT systems now employ more advanced reconstruction algorithms, including iterative reconstructions and artificial intelligence (AI)-based denoising, to enhance image quality in low-dose settings (see Fig. 1). These reconstructions, especially by AI, enable further dose reduction without compromising image quality and are, therefore, essential for ultra-low-dose scanning [6, 7].

While iterative reconstruction techniques often affect edge sharpness and are therefore less suitable in high-contrast environments—a key reason why societies still recommend filtered back projection for high-resolution CT of the lungs—AI-based reconstructions rely heavily on the training of their algorithms. Despite this caveat, AI reconstructions have shown significant potential for reducing radiation exposure, particularly in imaging the axial skeleton [8]. Radiation doses lower than those of standard radiography, while maintaining high image quality, are achievable, particularly for bone imaging, such as in the detection of erosions. Other applications have been described, e.g. for fracture detection [9] and 3D models [10]. This reduction in dose paves the way for new applications, including dynamic scans.

### Dynamic CT

CT systems with sufficient Z-axis coverage, such as volume detectors up to 16 cm or dual-source CTs, enable continuous scanning of an object without table movement, thereby providing additional temporal information [11]. Dynamic CT can thus offer twofold information: first, it can provide data on dynamic enhancement after contrast injection in non-moving structures, referred to as dynamic contrast-enhanced CT (DCE-CT); second, it provides information on the movement of bones relative to each other during dynamic motion [8, 12].

#### DCE-CT

DCE-CT entails the acquisition of multiple imaging scans concurrent with the intravenous administration of a contrast agent being administered intravenously. It enables quantification of joint perfusion by analyzing the temporal uptake dynamics of the contrast agent within the synovial tissue [13]. This methodology facilitates enhanced insights into potential variations in perfusion characteristics and offers a quantitative evaluation of the severity of inflammatory activity. Nevertheless, the clinical application of DCE-CT in the diagnostics of arthritis remains somewhat constrained. This limitation is partially attributable to the requirement for specialized acquisition protocols and dedicated software for post-processing measurements through region of interest (ROI) analysis. Thus, the utilization of DCE-CT is predominantly restricted to research environments, particularly for assessing therapeutic responses in inflammatory arthritis.

#### 4D-CT

4D-CT allows for rapid assessment of joint instabilities and can provide unique insights, such as those involving the distal radioulnar joint or dynamic carpal instabilities, which are challenging to assess using conventional methods [14]. However, it remains unclear which patients will benefit most from this assessment or whether its application will remain confined to research purposes. Beyond the wrist, other joints are also amenable to dynamic assessment, though applications in the context of inflammatory arthritides have not yet been published.

### Contrast-enhanced CT

Identifying active inflammation in joints and tendons is essential for diagnosing arthritis [15]. To visualize inflammatory changes in these structures, intravenous contrast agents are typically administered. Traditionally, CT has been viewed as less effective than imaging methods like ultrasonography or MRI for detecting active joint inflammation in clinical practices. This is due to issues such as image noise and variations in soft tissue density among individuals. However, MRI is not advised as the first imaging choice for early arthritis; it is reserved for cases where initial evaluations are inconclusive [16]. Notwithstanding these challenges, recent advancements in computed tomography (CT) technology have markedly enhanced the capacity to visualize active inflammation with greater efficacy [5, 13, 17]. One notable advancement in imaging technology is computed tomography subtraction (CT-S) imaging [5].

**Fig. 2:**
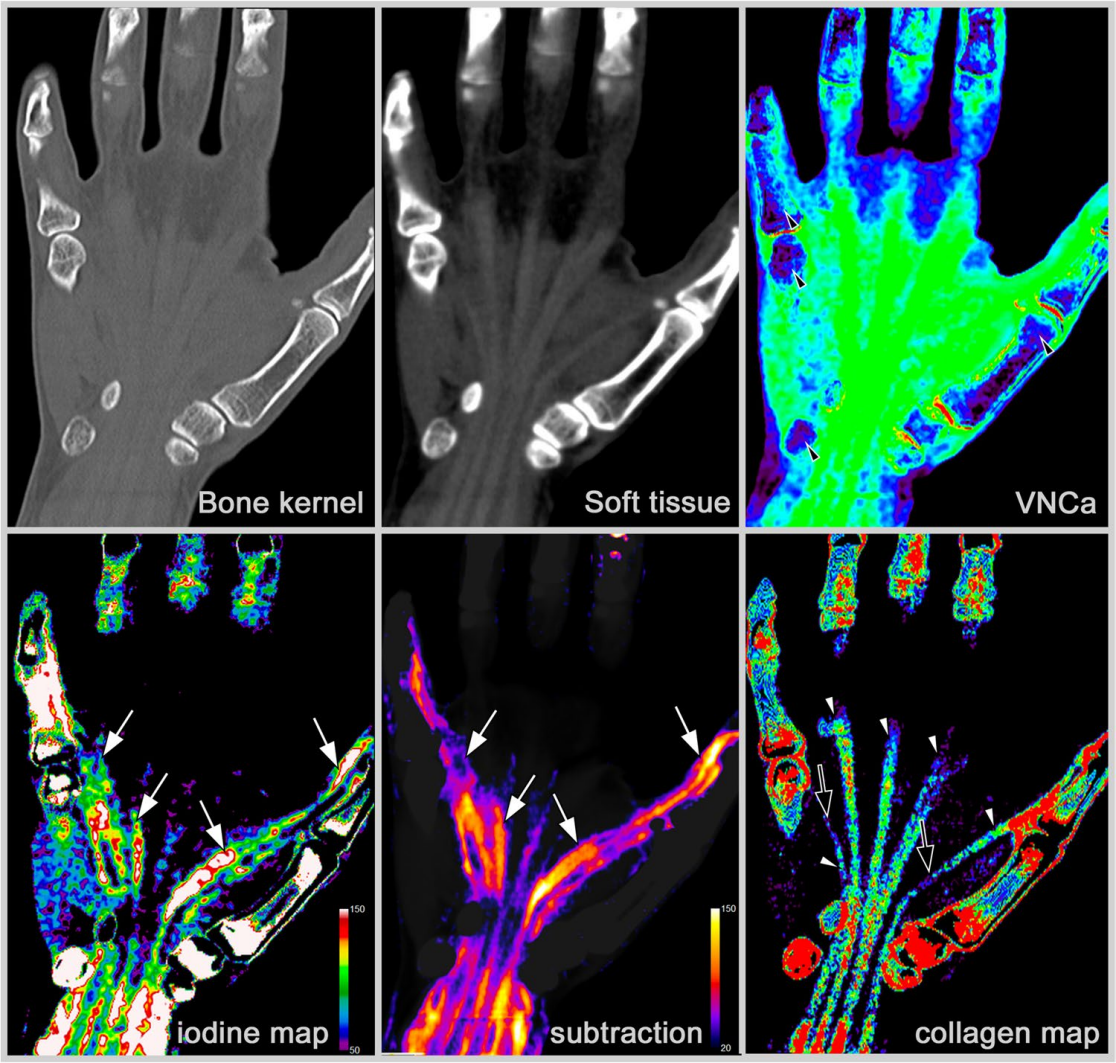
Reconstructions derived from contrast-enhanced DECT in a patient with rheumatoid arthritis. Presented are images in standard bone and soft-tissue kernel. Virtual non-calcium images (VNCa) show normal bone marrow (black arrowheads). Iodine maps show enhancement of the flexor tendons (white arrows); however, the inflammation is better depicted in CT subtraction (white arrows). Collagen maps show the flexor tendons (white arrowheads) with somewhat decreased collagen density in areas with marked inflammation (black arrows)

CT-S involves performing scans preceding and succeeding the intravenous administration of contrast media, followed by the subsequent subtraction of these scans. The resultant image delineates a pure contrast-enhanced visualization, thereby facilitating the evaluation of active inflammation (see for an example Fig. 2). It is imperative to recognize that the efficacy of CT-S is profoundly contingent upon patient compliance. Any motion occurring between the pre- and post-contrast scans can engender substantial artifacts, thereby complicating the interpretative process. In response to this challenge, most established reconstruction algorithms employed in clinical and research settings integrate motion correction techniques to mitigate such artifacts. CT-S affords a robust and reliable methodology for detecting active inflammation in joint structures, thereby endorsing its application not only in research contexts but also increasingly in standard clinical practice [5].

### Spectral CT

The advancement of conventional CT technology to spectral CT allows the acquisition of additional information through its versatile reconstruction algorithms. In dual-energy CT (DECT), two distinct CT datasets are acquired using different tube voltages [18]. This takes advantage of the fact that materials exhibit different attenuation coefficients at varying X-ray energies, enabling differentiation of tissues and materials that would be indistinguishable with conventional CT [18, 19]. A practical example of multiple reconstructions from DECT is shown in Fig. 2.

#### Two-material decomposition

One of the first clinical applications of DECT is the ability to differentiate two materials based on their attenuation coefficients, such as distinguishing uric acid from calcium, which plays a significant role in detecting gout and CPPD [20, 21]. This approach enables standardized and automated assessment of tophus burden, which is increasingly becoming an independent and relevant parameter for therapy monitoring and disease progression control in clinical practice [22].

### Three-material decomposition

An additional benefit of material-specific imaging is the implementation of three-material decomposition, which facilitates the precise identification of three distinct materials within a defined volume, taking into account their respective concentrations and partial volume effects. A pertinent example of this application is the visualization of bone marrow edema utilizing virtual non-calcium imaging (VNCa) techniques [23], where calcium, fat and water are the three primary components, with the calcium effect removed from the virtual image. In this subtracted image, an elevated water content in typically fat-rich bone marrow—often resulting from inflammation—signals the presence of bone marrow edema. The value of imaging bone marrow edema in inflammatory joint diseases is rooted in its ability to signify early irreversible structural changes, like erosions, emphasizing the need for prompt and precise detection. Recent years have seen a growing interest in leveraging VNCa for peripheral arthritis within research contexts, yielding promising initial outcomes that may translate into clinical practices [24, 25].

An additional application of three-material differentiation involves the depiction of active inflammation through the quantification of iodine concentration within the contrast medium [17, 26]. This methodology facilitates the generation of virtual iodine maps (iMaps), which effectively visualize active inflammation in joints and tendons. This technique is being increasingly employed in research studies that not only detect but also quantify iodine content, thereby offering deeper insights into perfusion characteristics [27, 28].

A particularly interesting application of three-material differentiation in the diagnostic workup of inflammatory joint diseases is collagen imaging. Collagen is an indispensable protein that significantly contributes to the architecture and stability of connective tissues, particularly tendons and ligaments. In individuals diagnosed with inflammatory joint diseases, the inflammatory processes may induce alterations in the structural integrity of ligaments and tendons, which are frequently correlated with variations in collagen density [29]. Understanding changes in collagen density is still under investigation, and while it remains an active area of research, its integration into clinical practice is yet to be discussed.

#### Virtual monochromatic images

In the context of inflammatory diseases, the use of virtual monoenergetic imaging (VMI) is primarily found in research settings [30]. VMI is an advanced technique in spectral CT that allows for the reconstruction of virtual images at a single, user-defined energy level, providing a clearer view of specific tissue characteristics [31]. It is based on the assumption that low-energy VMI simulates lower photon energies without the drawbacks of a broad X-ray spectrum, resulting in improved contrast between structures with different effective atomic numbers (Zeff) [32]. Applied to inflammatory joint diseases, this can be observed in the case of iodinated contrast agents and soft tissues. Studies have shown that VMIs can detect inflammation with sufficient diagnostic accuracy, but the improved contrast is offset by higher noise levels, which provides no discernible advantage for quantitative or subjective evaluation [30].

### Photon-counting CT

A recent advancement in CT detector technology, photon counting detectors, holds significant potential for improving CT imaging. Photon-counting detectors are more sensitive to X-rays than conventional energy-integrating detectors and are inherently sensitive to photon energy, enabling the acquisition of spectral information in every scan [33]. As a result, they offer higher spatial resolution, reduced radiation exposure, and enhanced reconstructions, allowing for the detection of bone marrow edema or gouty tophi [34].

Currently, small scanners for extremity imaging are available from various manufacturers, but only one vendor has produced a whole-body scanner, and data on its performance for arthritis assessment are not yet available. On one hand, improved spatial resolution with photon counting technology is expected to enhance arthritis imaging, potentially detecting smaller erosions, similar to high-resolution peripheral quantitative CT [35]. On the other hand, the quality of spectral separation and information remains uncertain, raising the question of whether dual-energy data—i.e., scanning with two different energy levels—will continue to be preferred over spectral information derived solely from the photon-counting detector. However, dual-energy scanning with a photon-counting detector might be an ideal and possible solution.

### CT-like imaging with MRI

CT, particularly spectral CT, has established its utility for imaging arthritis in both the peripheral and axial skeletons. Concurrently, MR-based imaging techniques have progressed to produce images that closely imitate the osseous detail provided by CT, while retaining the unique advantages inherent to MRI. Therefore, advanced methods, such as zero echo time (ZTE) imaging, susceptibility-weighted imaging (SWI), volumetric interpolated breath-hold examination (VIBE), and synthetic CT reconstruction from MRI data, have been developed.

ZTE MRI offers the distinct advantage of direct visualization of bone by capturing signals from short-T2 tissues, making it particularly effective in assessing cortical bone and identifying erosive changes associated with inflammatory arthritis. Research has substantiated its potential as an alternative to conventional CT imaging, particularly within pediatric populations [36]. SWI enhances the visualization of bone marrow pathologies and calcifications by utilizing phase differences in tissue susceptibility. Although it does not match the high-resolution osseous details obtained from CT, SWI enhances specificity in the detection of erosions [37].

VIBE, a gradient echo-based MRI sequence, generates high-resolution three-dimensional reconstructions that simulate CT images, thus facilitating osseous evaluation. This technique has been employed in musculoskeletal imaging to assess structural damage associated with arthritis, striking a balance between bone and soft tissue contrast. While VIBE, as a rapid gradient echo sequence, is potentially subject to non-desired T2* effects, findings have indicated that it may be less optimal for direct bone depiction in peripheral arthritis [38]. Recent advancements in artificial intelligence, including deep learning and machine learning, have enabled the generation of synthetic CT images from MRI sequences [39].

Adding MR-based CT-like images to the standard MR protocol presents notable advantages over conventional CT, such as enhanced soft tissue contrast, the absence of ionizing radiation, and the capability for multi-contrast evaluations. While CT remains the gold standard for meticulous osseous assessment, MR-based imaging strategies may serve as viable alternatives in specific clinical circumstances, particularly when radiation exposure is a crucial concern.

## Peripheral arthritis

### Gouty arthritis

CT, particularly spectral CT, is recognized as a standard imaging modality for the assessment of gouty arthritis and is included in the diagnostic criteria for gout due to its superior diagnostic performance, with reported sensitivity and specificity of 92% and 89% [3, 40, 41]. In this context, DECT can not only identify the depositions of monosodium urate within gouty tophi but also delineate structural lesions and bone marrow edema [42]. Furthermore, it can potentially eliminate the need for joint aspiration based on its findings. However, there is currently no consensus regarding the optimal imaging protocol, particularly in terms of which joints should be included [43]. Institutional standards vary from limited protocols that include only one clinically affected joint to comprehensive systemic stagings that incorporate all major peripheral joints. Research has demonstrated that the prevalence of tophi is highest in the feet and ankle joints, followed by the knees and elbows [44]. Consequently, it is reasonable to include scans of the ankles and forefeet in all assessments for gouty arthritis, even when the clinical presentation is limited, e.g., to the hands.

**Fig. 3:**
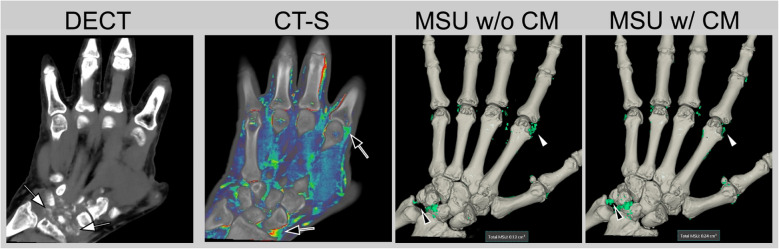
Unenhanced and enhanced DECT of a patient with gouty arthritis. This patient presented with a clinical suspicion of psoriatic arthritis, supported by normal radiographic findings of the hands. Dual-energy computed tomography (DECT) demonstrated the presence of crystal deposits in the wrist and metacarpophalangeal joints (white arrows), alongside enhancement post-contrast injection (black arrows). The volumetric assessment of monosodium urate (MSU) reconstructions was shown to vary based on pre-contrast (w/o CM) and post-contrast (w/CM) scans, with certain localizations exhibiting greater MSU volume (black arrowheads) while others displayed reduced volumes (white arrowheads)

The sensitivity of DECT for the early diagnosis of gout is notably limited due to the requirement that a tophus must be present with a density and size that exceeds the detection threshold established by the algorithm [45]. The density of tophus is quantitatively correlated with the concentration of monosodium urate within the voxel [46]. In patients diagnosed with early-stage gout, the presence of a double-contour sign observed on ultrasound, in the absence of tophus formation, typically results in negative findings on DECT [47]. Therefore, non-tophaceous gout will not be detected by DECT. Furthermore, it is crucial to recognize that the application of contrast media can significantly influence the assessment of tophi, altering sensitivity based on the degree of enhancement observed (see Fig. 3) [48, 49]. Various methodologies, particularly distinct postprocessing techniques, have been implemented to augment the sensitivity of DECT; however, these efforts have generally resulted in only modest improvements [50–52]. Consequently, it remains imperative for radiologists to conduct a thorough evaluation of the imaging findings for additional indicators of inflammatory arthritis, particularly the presence of erosions and bone marrow edema.

### CPPD

**Fig. 4:**
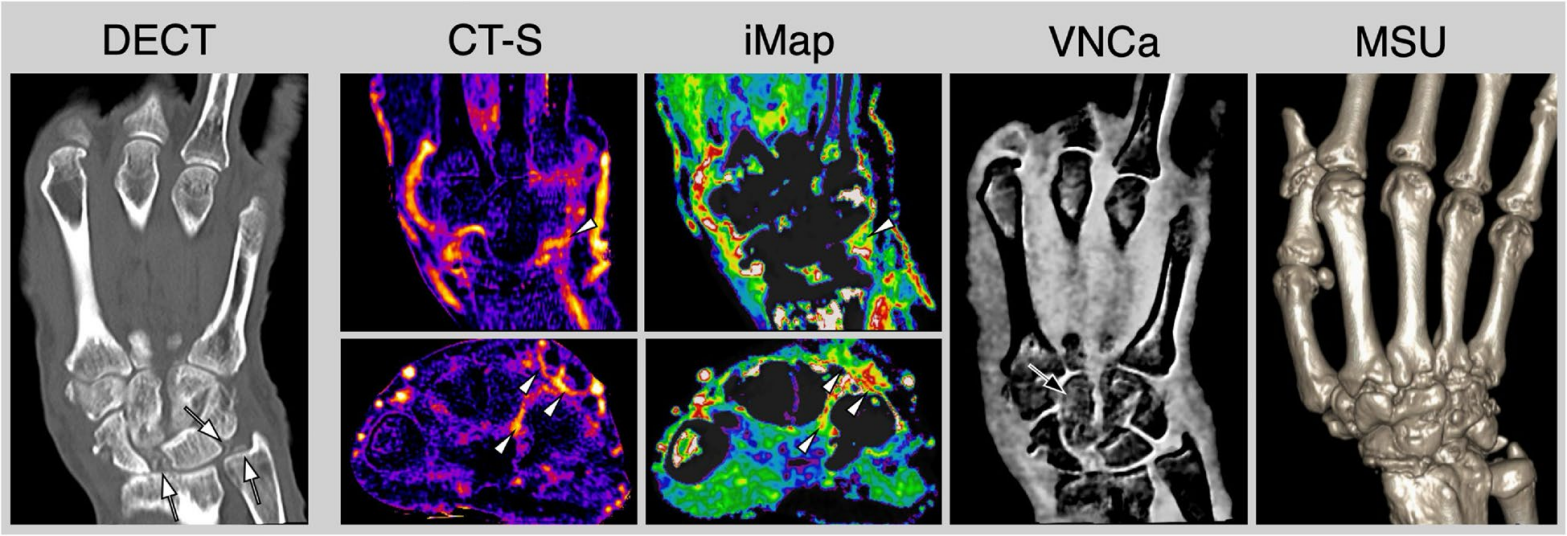
Multiparametric contrast-enhanced dual-energy computed tomography (DECT) in a patient with calcium pyrophosphate dihydrate (CPPD) crystal deposition disease. Conventional DECT revealed the presence of crystal deposits in the scapholunate and lunotriquetral ligaments, as well as in the triangular fibrocartilaginous complex (white arrows). Furthermore, contrast-sensitive reconstructions using computed tomography subtraction (CT-S) and iodine maps derived from DECT (iMap) demonstrated significant active synovitis and tenosynovitis (white arrowheads). Virtual non-calcium images (VNCa) illustrated mild bone marrow edema localized in the capitate bone (black arrow). Notably, the crystals were found to be negative for monosodium urate (MSU). In conclusion, multiparametric CT helped in establishing a definitive diagnosis of active CPPD

Calcium pyrophosphate dehydrate deposition disease (CPPD) is characterized by the accumulation of calcium pyrophosphate dihydrate crystals in joint tissues, most notably in the ligaments of the wrist. This accumulation leads to inflammation and ligament damage, with interleukin- 1β playing a key role in the pathophysiological processes [53]. CT and especially DECT have emerged as a significant imaging modality in the diagnosis and management of CPPD [54]. Due to its high spatial resolution, DECT can accurately identify calcifications associated with CPPD and differentiate between various types of crystalline deposits [55]. Specifically, DECT utilizes two-energy levels to effectively differentiate between various materials, leveraging their unique attenuation properties. This capability facilitates the identification of calcium pyrophosphate dihydrate (CPPD) crystals, allowing for the discrimination of subtle calcifications that remain undetectable by conventional imaging modalities, such as radiography and ultrasonography. The detection of ligament calcifications associated with CPPD poses significant challenges, particularly within anatomically intricate regions that are often inaccessible to traditional imaging techniques. A notable example is the palmar carpal ligaments, located in the deeper structures of the wrist joint, which exemplify this diagnostic difficulty [55] and are often hard to assess using standard X-ray or ultrasound imaging due to their positioning and the limitations of these modalities. Moreover, the capability of DECT to detect early and subtle calcifications prior to the onset of significant joint damage presents a substantial opportunity for enhancing the management of CPPD and offers a non-invasive and highly effective method for the early diagnosis and continuous monitoring of disease progression (see Fig. 4). Consequently, the importance of DECT in the diagnostic framework is duly acknowledged by the 2023 European League Against Rheumatism (EULAR) imaging recommendations pertinent to crystal arthropathies [54].

Initial DECT studies have demonstrated that CPPD deposition can alter the biomechanics of the carpal ligament apparatus [56, 57]. Dynamic CT of the wrist can reveal sequelae in the form of ligament tears, typically occurring at the scapholunate junction or distal radioulnar joint [58]. These ligament tears are precursors to subsequent joint degeneration, contributing to the characteristic pattern seen in advanced cases of these diseases.

### Other inflammatory arthritides

The utilization of CT and DECT has been established as essential in the evaluation and management of various forms of inflammatory arthritides, extending beyond the previously mentioned crystal arthropathies. Recent advancements in technology, characterized by their diverse capabilities, hold significant potential to broaden the application of CT in the assessment of inflammatory joint diseases. CT facilitates the identification of imaging-based morphological parameters that are pertinent to disease characterization. For instance, in the context of rheumatoid arthritis (RA), which has an approximate global prevalence of 0.24% [59] and represents the second most common inflammatory joint disease after gout, CT can make a significant contribution. In addition to its capacity for reliably detecting structural lesions, including erosions and ankyloses, CT, particularly through its multiparametric approaches, enables the visualization of synovitis, tenosynovitis, and bone marrow edema. Furthermore, CT is extensively employed in the research of rheumatoid arthritis (RA), yielding promising outcomes and demonstrating non-inferiority in comparison to MRI [17].

**Fig. 5:**
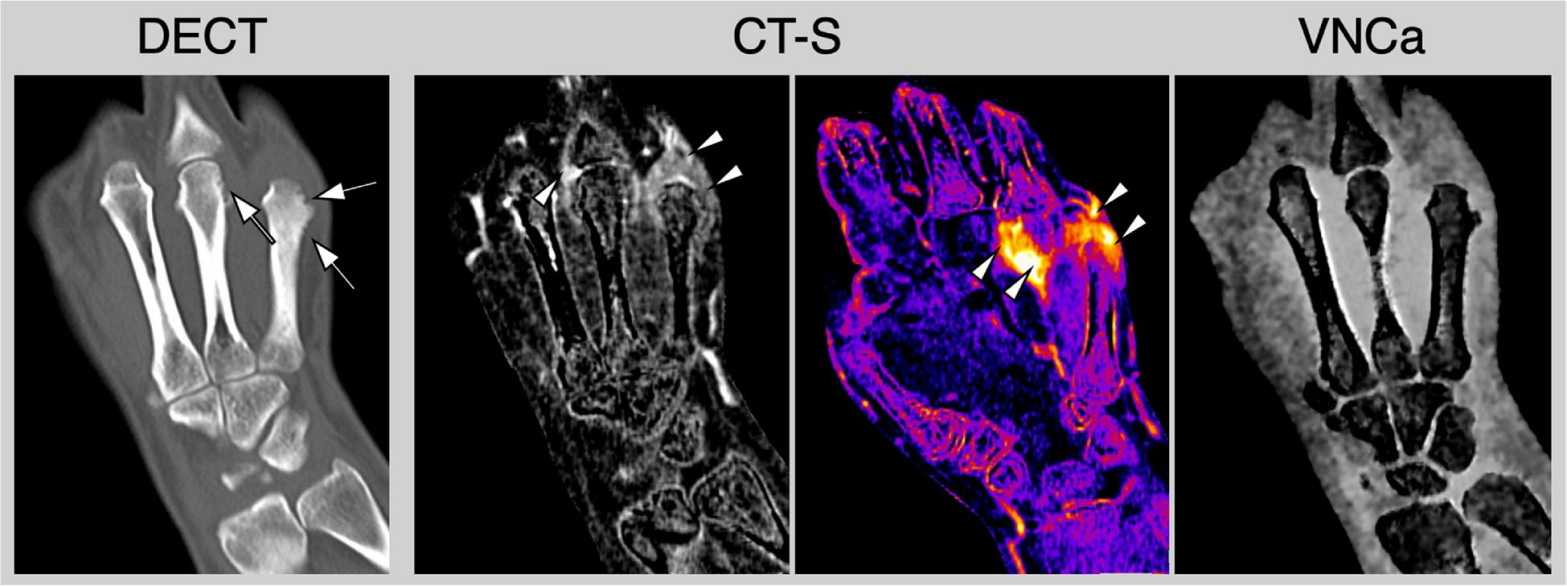
Multiparametric computed tomography in a patient with psoriatic arthritis. Conventional DECT imaging demonstrates both erosive changes and the presence of new bone formation (white arrows). The CT-S in greyscale and color-mapping techniques identifies active synovitis in the fourth and fifth metacarpophalangeal joints (white arrowheads). Additionally, VNCa does not show evidence of bone marrow edema in this patient who is diagnosed with active and erosive psoriatic arthritis

In psoriatic arthritis (PsA), CT is critical in identifying subtle bone changes, such as periosteal proliferation (see Fig. 5) [60]. Periosteal reaction at the enthesis, characterized by new bone formation at the margins of affected bones, is a hallmark finding in PsA [61]. This is particularly relevant because periosteal reactions can be challenging to detect with conventional radiography, especially during early disease stages or in anatomically complex areas. The high resolution of DECT further enhances the assessment of other key structural changes associated with PsA, such as enthesitis (inflammation at tendon or ligament attachment sites) and synovitis, providing valuable insights into disease diagnosis and progression. The DECT-specific reconstruction algorithms described above contribute to the reliable detection of these pathological changes, making CT an increasingly valuable tool in both research and clinical practice.

## Axial spondyloarthritis

**Fig. 6:**
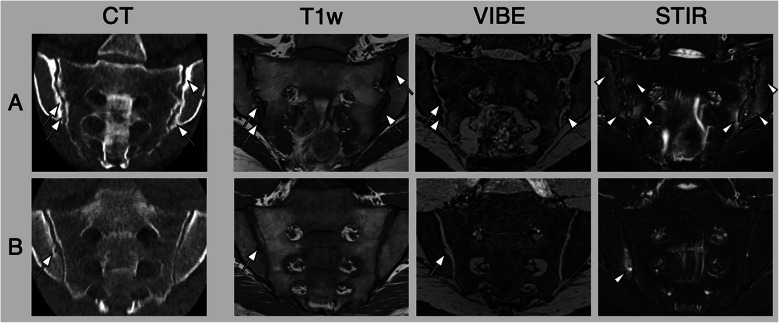
Low-dose CT in patients with axial spondyloarthritis. Both scans were conducted utilizing a volume scan technique without table movement, employing parameters of 120 kVp and 5 mAs. This methodology resulted in a dose length product of 2.6 mGy*cm and a computed tomography dose index volume (CTDIvol) of 0.3 mGy, with an estimated effective dose of less than 0.04 mSv. The reconstruction of the scans was performed using artificial intelligence algorithms. Patient A displays advanced sacroiliitis characterized by bilateral erosion and sclerosis (white arrows) along with bone marrow edema (white arrowheads). Conversely, patient B exhibits only mild erosion (white arrow) and bone marrow edema (white arrowhead)

The assessment of axial spondyloarthritis (axSpA) involves imaging two regions of the axial skeleton. For primary diagnosis, the sacroiliac joints (SIJ) are typically the focus of imaging, whereas follow-up during treatment, particularly in advanced cases, shifts the focus to the spine. The advent of ultra-low-dose protocols has made CT a feasible imaging option even in younger patients, especially when it provides more information with less radiation compared to radiography [62]. Current ultra-low-dose protocols using modern reconstruction techniques push the limits of scanner hardware in terms of minimizing radiation, and there are still developments needed from manufacturers to optimize scan planning and execution to fully utilize the potential of radiation reduction (see Fig. 6).

**Fig. 7:**
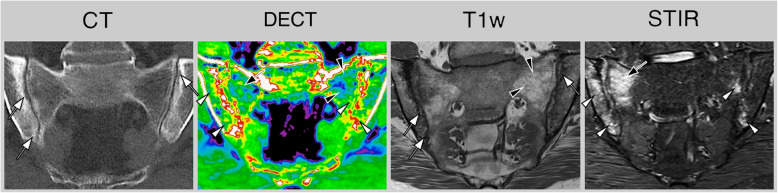
Dual-energy computed tomography (DECT) of a patient with axial spondyloarthritis. Computed tomography (CT) and T1-weighted imaging reveal evidence of erosion (white arrows) and sclerosis. DECT shows increased density in regions characterized by bone marrow edema (white arrowheads) as well as decreased density in adipose tissue lesions (black arrowheads). Additionally, there is a notable mixed density observed in a specific region exhibiting bone marrow edema within a fat lesion (indicated by a black arrow)

Another promising development is spectral CT, which offers the possibility to evaluate bone marrow in axSpA patients for signs of bone marrow edema or fat lesions [62–64]. While this is an intriguing prospect that could combine excellent structural assessment with sensitivity for active inflammation, it does not align with the goal of minimizing radiation exposure. On the contrary, published protocols have radiation doses comparable to, or even exceeding, those of standard abdominal CT. This technique is also prone to false-positive findings in cases of reduced fat within the bone marrow, such as when marked sclerosis is present [65]. Furthermore, while spectral CT performs reasonably well in diagnosing osteoporotic fractures in an elderly population, the higher proportion of red bone marrow (and correspondingly lower yellow marrow and fat content) necessitates an increased Hounsfield Unit threshold for detecting bone marrow edema in young axSpA patients, which in turn reduces sensitivity for said lesions. As such, the application of dual-energy CT (DECT) for the SIJ or spine in axSpA remains a topic of debate in clinical practice (see Fig. 7 for an example).

### Sacroiliac joints

For the SIJ, the detection of lesions is crucial for objectifying inflammation and establishing a diagnosis of axSpA, and the absence of lesions is usually sufficient to rule out sacroiliitis [4]. In this context, joint erosion, similar to other forms of arthritis, serves as a significant indicator due to its high sensitivity and specificity [66]. While conventional and low-dose CT are not sensitive to active bone marrow inflammation, which is the cornerstone of a positive MRI according to the Assessment of SpondyloArthritis international Society (ASAS) criteria, it is important to emphasize that these criteria were not designed for routine clinical assessment or diagnostic confirmation [67]. In clinical practice, the presence of structural lesions such as erosion, ankylosis, and backfill (less so sclerosis) is at least equally, if not more, important than bone marrow edema [68]. Therefore, CT might be a practical diagnostic imaging modality for the assessment of axSpA.

In terms of diagnostic performance, CT demonstrates reasonably high sensitivity and very high specificity compared to MRI and radiography, and adding CT to MRI can increase the overall specificity of the assessment [4]. Therefore, CT is a viable alternative cross-sectional imaging method, particularly when MRI is unavailable, or the patient is unwilling to undergo an MRI scan. It is also an excellent complementary imaging technique when MRI results are inconclusive or when the MRI protocol is insufficient.

Recent studies using CT to examine the SIJ highlight an important aspect: CT is the first imaging technique capable of identifying variations in joint shape that can affect mechanical load [69]. These variations may lead to bone marrow edema or sclerosis, which can mimic sacroiliitis, especially when assessed with MRI [70]. While some of these shape variations can also be detected with standard MRI, CT’s superior spatial resolution and bone contrast make it the preferred method for this evaluation. Recognizing these joint shape variations is essential for differentiating true sacroiliitis from other causes of bone marrow edema, and CT might be utilized whenever such a condition is suspected on MRI.

Attempts have been made to train AI algorithms to detect erosion and sclerosis at the SIJ, with corresponding studies showing moderate diagnostic accuracy [71]. However, since CT is not a first-line imaging modality and young patients do not routinely undergo CT scans that could be used for opportunistic screening, the clinical impact of these algorithms remains uncertain. These software approaches may be useful for identifying sacroiliitis in patients with inflammatory bowel disease, although it should be standard practice to evaluate the SIJ in these cases. In the future, automated grading or scoring of lesions could facilitate clinical studies or be used for routine follow-up scans.

### Spine

For the spine, studies have demonstrated that CT is more sensitive to new bone formation, such as syndesmophytes, compared to MRI and radiography [72]. MRI often fails to depict black cortical bone within the black ligamentous environment of the anterior and posterior longitudinal ligaments, while radiography suffers from superposition, particularly at the thoracic spine. However, the thoracic spine is often the site of most lesions. CT also excels in assessing structural lesions of the small spine joints, such as facet joints or costovertebral joints, due to its high spatial resolution and superposition-free imaging [73].

As a result, CT enables a swift and highly accurate assessment of structural spine lesions, which can shorten the observation period for clinical studies [74]. Overall, CT could be considered the gold standard for assessing structural spine lesions, although it is not routinely used in clinical practice. Nevertheless, CT has the potential to revolutionize clinical trials and enhance clinical practice as soon as structural progression monitoring is incorporated into routine follow-up, which is currently only performed clinically [75].

**Fig. 8:**
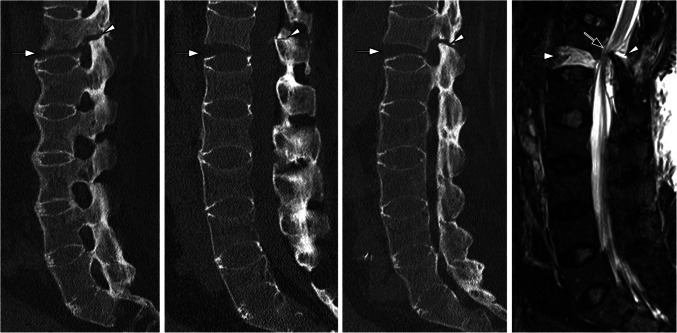
A patient with advanced axial spondyloarthritis and minor trauma. The patient presents with a vertebral column fracture at the D12 level (white arrow) accompanied by involvement of the posterior elements, as denoted by the white arrowheads, resulting in an unstable condition. Magnetic resonance imaging (MRI) reveals a corresponding spinal canal stenosis (black arrow) that is not clearly assessable with CT

Patients with ankylosing spine disease, such as axSpA or diffuse idiopathic skeletal hyperostosis (DISH), should undergo CT imaging of the spine whenever a fracture is suspected. Ankylosis of the spine and osteoporosis as a comorbidity make these patients prone to unstable vertebral column fractures (so-called chalk stick fractures), even with minor trauma (see Fig. 8). CT plays a critical role in diagnosing these fractures. Therefore, the indication for cross-sectional imaging, particularly CT, should be made early and without hesitation.

## Conclusion

CT has long been regarded as a niche modality in arthritis imaging. However, developments such as ultra-low-dose protocols and spectral information make CT a viable alternative to other cross-sectional imaging methods for arthritis assessment. While CT may lack the sensitivity for soft-tissue and bone marrow inflammation compared to MRI, it provides high specificity through superior detection of cortical and trabecular bone changes, thus offering reliable information about structural lesions. It can also identify differential diagnoses that are difficult to detect on MRI, such as crystal disease or anatomical variations. In conclusion, CT is a modality that warrants further research and could see broader application in clinical practice today.

## Data Availability

Not applicable.
